# Achieving Operational Hydrologic Monitoring of Mosquitoborne Disease

**DOI:** 10.3201/eid1109.050340

**Published:** 2005-09

**Authors:** Jeffrey Shaman, Jonathan F. Day

**Affiliations:** * Oregon State University, Corvallis, Oregon, USA;; † University of Florida, Vero Beach, Florida, USA

**Keywords:** hydrology, mosquitoes, mosquitoborne disease, monitoring, forecasting, transmission, amplification, synopsis

## Abstract

Mosquitoes and mosquitoborne disease transmission are sensitive to hydrologic variability. If local hydrologic conditions can be monitored or modeled at the scales at which these conditions affect the population dynamics of vector mosquitoes and the diseases they transmit, a means for monitoring or modeling mosquito populations and mosquitoborne disease transmission may be realized. We review how hydrologic conditions have been associated with mosquito abundances and mosquitoborne disease transmission and discuss the advantages of different measures of hydrologic variability. We propose that the useful application of any measure of hydrologic conditions requires additional consideration of the scales for both the hydrologic measurement and the vector control interventions that will be used to mitigate an outbreak of vectorborne disease. Our efforts to establish operational monitoring of St. Louis encephalitis virus and West Nile virus transmission in Florida are also reviewed.

## Abstract 2

West Nile virus transmission in Florida can be monitored by using modeled hydrology.

Operational systems that can accurately monitor and predict mosquitoborne disease transmission continue to be needed. Because mosquitoes and the pathogens they transmit are sensitive to environmental conditions, 1 approach has been to use our ability to monitor and predict environmental variability and our understanding of mosquito and mosquitoborne pathogen response to that variability to monitor and predict mosquitoborne disease transmission. This reasoning assumes that we can monitor and predict environmental variability over large areas at the scale at which it affects mosquitoborne disease transmission and that the relationships between environmental variability and mosquitoborne transmission response are clear and stationary. Here we review the recent developments of operational systems that use hydrologic variability to monitor mosquitoborne disease transmission.

## Rainfall and Mosquitoborne Disease

Many mosquito species depend on the availability of water. The first 3 stages of the mosquito life cycle (egg, larvae, and pupae) are aquatic. Consequently, mosquito abundance and the transmission of many mosquitoborne pathogens can be affected by hydrologic variability, in particular, fluctuations in the water cycle that alter the availability of suitable aquatic habitats. To explore these effects, researchers have long looked for associations between rainfall variability and mosquito abundance (*1**–**4*) and mosquitoborne disease incidence (*5**–**12*). Although using rainfall as an explanatory hydrologic variable is convenient, the physical effects of precipitation on surface conditions are multiple, and the responses of different mosquitoes and mosquitoborne pathogens to these effects are varied. As a result, establishing statistically significant and stationary relationships between precipitation and mosquito abundance or mosquitoborne disease transmission is difficult.

Rainfall has 2 principal influences on the mosquito life cycle: 1) the increased near-surface humidity associated with rainfall enhances mosquito flight activity and host-seeking behavior, and 2) rainfall can alter the abundance and type of aquatic habitats available to the mosquito for oviposition. The first influence can increase mosquito abundance by accelerating the reproductive cycle, which requires mating, host-seeking, and blood-feeding flights. The second influence, however, has less certain consequences. Rainfall increases the wetness of soil near the surface and can expand saturated lowland areas. As a result, the moist, humid habitats preferred by many mosquito species for oviposition, such as swamps and floodwaters (e.g., puddles, water-filled divots), may increase in abundance. This change may favor an increase of mosquito species abundance in these habitats. Such changes in mosquito species composition, abundance, and age structure may then lead to an increase in local disease transmission.

However, the availability of suitable mosquito habitats is not a simple linear function of rainfall. Surface wetness depends on a number of environmental conditions other than precipitation, including antecedent wetness, soil type, and rates of evapotranspiration (i.e., combined evaporation and transpiration). Furthermore, excessive rainfall can decimate some mosquito populations by flushing larval habitats. Other mosquito species can benefit from drought conditions such as when streams dry up and pools more suitable for oviposition form in riverbeds, or when standing waters become eutrophic with increased organic content, which provides additional food for mosquito larvae.

Further complications arise when attempts are made to associate rainfall with mosquitoborne disease incidence. Mosquitoborne disease transmission is related most directly to the number of infected mosquitoes able to transmit disease and not to the total number of biting mosquitoes present in a population (*13**,**14*). As a consequence, increased mosquito abundance does not necessarily increase mosquitoborne disease transmission. As mosquito abundance increases, mosquito infection rates must also increase if disease transmission risk is to increase substantially; this requires that newly emerged mosquitoes acquire pathogens and become infective.

## Monitoring Mosquito Breeding Habitats

The response of mosquito populations to changes in precipitation and the effects of such changes on mosquitoborne disease transmission are quite complex and variable. However, these responses might be better elucidated if mosquito-breeding habitat availability, the variable for which precipitation is principally a proxy, could be monitored directly.

Several studies have examined how water management practices (e.g., irrigation, damming) affect anopheline density and malaria incidence. Surface waters on rice-cultivated land were associated with *Anopheles gambiae* density in the Ivory Coast (*15*); anopheline densities in Thailand were associated with rice paddy fields (*16*); and in Peru, irrigation around villages and houses played a role in determining human malaria risk (*17*). In Tanzania, *An. arabiensis* densities were 4 times higher in villages with rice cultivation, but malaria exposure was lower because of greatly decreased sporozoite rates among this mosquito population (*18*). This last study again illustrates some of the complexity underlying the relationship between mosquito abundance and disease transmission risk.

Although the effects of irrigation and water control are clearly important, for many disease systems natural surface water variability is likely an even greater determinant of vector density and mosquitoborne disease transmission rates. However, because monitoring surface water has traditionally been difficult, relatively few studies have explored these relationships. In Uganda, malaria incidence among children was associated with the proximity of their homes to swamps and streams that served as mosquito breeding sites (*19*). In Sri Lanka, the abundance of the primary malaria vector, *An. culicifacies*, has been linked to the drying of riverbeds (*20**,**21*).

Because ground observations of surface water prevalence are time-consuming and difficult to carry out over large areas, an attractive alternative has been to use remote sensing measurements of land surface wetness. Many such top-down studies have associated the abundance of vectors or vectorborne disease incidence by using satellite imaging (*22**–**28*). Such investigations have generally used vegetation classification or the Normalized Differential Vegetation Index (NDVI), which measures vegetation greenness, as proxies for soil moisture and land surface wetness.

A more recent approach has been to simulate land surface wetness conditions by using a hydrology model. Such models can represent the hydrologic cycle at the land-atmosphere interface and track the movement of water and energy between the soil, vegetation, and atmosphere. By accounting for soil type, vegetation type, topography, evapotranspiration rates, and precipitation, one may continuously simulate representation of surface pooling in space and time.

In some sense, the use of a hydrology model is a hybridization of bottom-up (ground observation) and top-down (satellite imaging) approaches and can be developed in conjunction with both through data assimilation. This approach has several advantages: 1) it models the actual aquatic environment used by the mosquitoes, not a filtered proxy; 2) it offers continuous real-time prediction of hydrologic conditions, that is unconstrained by orbital patterns, cloud cover, or vegetation; 3) it resolves the whereabouts of the potential breeding habitats at a very fine scale (areas as small as 10-m cells); and 4) hydrologic models are readily coupled to global climate models, allowing additional medium- and long-range forecast of hydrologic conditions.

Several studies have employed such models. Patz et al. (*29*) used a water balance model to hindcast weekly soil moisture levels in the Lake Victoria basin. These soil moisture levels were then associated with local human biting rates and entomologic inoculation rates. Shaman et al. (*30*) used a more detailed hydrology model to predict flood and swamp water mosquito abundances in New Jersey. Mosquito species were found to respond differently to changing local wetness conditions, and these differences were consistent with known breeding behavior and habitat preferences. For example, swamp and flood water mosquito abundance increased during wet conditions, while mosquitoes that preferred eutrophic breeding habitats increased during dry periods. Thus, hydrologic variability was able to differentially predict mosquito species abundances. In a separate study in Florida, amplification and transmission of St. Louis encephalitis virus (SLEV) were associated with changing modeled land-surface wetness conditions (*31*).

## Developing Early Warning Systems

Irrespective of the hydrologic variable measured (e.g., measured rainfall, water management, or irrigation effects; satellite measurements of land-surface wetness; or modeled hydrologic conditions), if the variable is associated with mosquitoborne disease transmission in a stationary and robust manner, it may be used to monitor that disease. A few such monitoring systems have recently been developed in which climatic conditions (monthly rainfall and temperature) appropriate for mosquitoborne disease transmission are used to develop risk maps of the geographic distribution of the disease. In Africa this risk assessment has been applied on a large scale and used to develop maps of malaria risk and distribution (*32**,**33*). Where precipitation is found to precede malaria transmission and the data are readily available, these maps could be used as part of a malaria early warning system. Such maps are an important step toward achieving operational monitoring and forecasting of malaria transmission.

## Issues of Scale

Before hydrologic factors are used to monitor and forecast mosquitoborne disease transmission, the scales at which the disease system responds to hydrologic variability, as well as the scales at which hydrologic variability can be monitored, must be considered. For instance, empirical findings may demonstrate that a particular disease vector responds to local variations in precipitation (i.e., hydrologic changes in its immediate environment). To monitor this vector population, one would like to keep informed of local rates of rainfall, preferably over the entire geographic range of the vector. However, in the tropics, precipitation rates can differ greatly between locations just a few kilometers apart, but meteorologic stations are much more sparsely distributed. The mismatch between the scales at which a disease vector responds to hydrologic variability and the scales at which hydrologic variability can actually be monitored limits operational application of such empirical findings and underscores the need to develop systems that monitor and forecast hydrologic variability at scales corresponding to disease system ecologies.

Operational monitoring and forecasting of any vectorborne disease also requires consideration of how information is to be used. Empirical relationships derived from either microenvironment- or macroenvironment-level scales must be relevant at the scales at which vector control interventions are applied. For example, modeling and mapping of individual mosquito oviposition sites (e.g., herbivore hoof prints) may prove too detailed, heterogeneous, and computationally expensive to be of practical use as an aid for vector control efforts. However, if the flooding and drying patterns of such hoof prints are highly covariable over a large spatial scale, for example, several square kilometers, then modeling flooding and drying at these sites might prove feasible. That is, rather than attempt to monitor and control oviposition sites individually, the herbivore hoof prints would instead be monitored collectively on a larger spatial scale. This information could then be used to help make vector-control intervention decisions at that larger spatial scale by focusing the application of larvicide to selected areas wet enough to support many hoof print pools. This approach would reduce unneeded control efforts in regions too dry to support such pools.

Similarly, seasonal climate model predictions may lack the temporal and spatial resolution needed to discern local disease transmission patterns. Resources may be wasted if control efforts are blanketed over a large region of which only small portions are "hot zones" of vector and disease activity. Yet if stable relationships exist between grid-scale and subgrid scale variability, useful information for intervention might be gleaned from such models. For instance, climate models often have a resolution (i.e., grid scale) of 2.5°×2.5°, about 75,000 km^2^. This is a very large area over which to adopt a single vector-control intervention strategy. Clearly, variable levels of vector and disease activity will exist within such an area. Local understanding of subgrid scale (i.e., scales <75,000 km^2^) variability and how it relates to grid-scale variability is needed to focus control efforts. This understanding could be as simple as knowing that during drought the rivers within a given 2.5°×2.5° area pool and serve as mosquito-breeding sites and that control efforts should be focused in the pools that form along these rivers.

Thus, the issue of scale has to be considered from both scientific and operational vantages. Not only must empirical relationships between mosquitoborne disease systems and hydrology be robust, they must also be capable of being monitored and used with vectorborne disease intervention programs. Monitoring hydrologic variability must be possible both at the scale at which it affects mosquitoes and mosquitoborne disease transmission as well as at a scale at which interventions can feasibly and effectively be applied. Such considerations, of course, are inextricably linked to an understanding of the biology and ecology of vectorborne disease systems.

## Beginning Operational Application: Florida

We now present an example from our own efforts to establish, real-time operational hydrologic monitoring of West Nile virus (WNV) in Florida. We begin with the scientific basis for this system then describe operational application of the system in 2004. Further development of the monitoring system is then discussed, and issues of scale are considered.

Since its appearance in New York City in 1999, WNV has spread to every state in the continental United States and has emerged as an important infectious disease threat to the general public. WNV was first detected in Florida in 2001; since then human cases of WNV illness have occurred sporadically throughout the state. WNV is closely related to SLEV. Both WNV and SLEV are maintained in the environment through enzootic transmission between avian amplifying hosts and vector mosquitoes (*34*). In a series of articles, we have demonstrated that a specific sequence of hydrologic conditions, spring drought followed by continued summer rainfall, is critical for the amplification and transmission of both SLEV and WNV in south Florida (*31**,**35**–**38*). Amplification involves a cascade of enzootic virus transmission between vector mosquitoes and wild avian hosts and results in a rapid increase in the number of infected and infective vector mosquitoes. Drought enhances amplification by restricting vector mosquito activity to selected habitats in south Florida, specifically densely vegetated hammocks where mosquitoes rest and wild birds nest and roost. Increased contact between vector mosquitoes and avian amplification hosts in these hammocks facilitates early season viral amplification by forcing the interaction of mosquitoes and birds.

When drought-induced, early season viral amplification in the initial transmission foci is followed by high amounts of summer rainfall that increases near surface humidity levels and the availability of oviposition sites, infective mosquitoes are able to disperse and initiate secondary transmission foci away from the original amplification site. This dispersal and secondary amplification in urban and suburban habitats dramatically increase the number of infective mosquitoes in the environment and place them into greater contact with humans during the late summer months, when WNV and SLEV epidemics typically occur in south Florida. In addition, the mosquito population can increase dramatically in response to the increased water resources. Because of the increased viremic rate among the wild bird population, these newly emergent mosquitoes are themselves more likely to acquire infection.

Our analyses cited above demonstrate that high transmission rates of SLEV and WNV to humans are more likely to occur after a spring drought that is followed by continuous summer rainfall. Public health and vector control personnel are greatly concerned about the likelihood of a major WNV epidemic in Florida. A widespread spring drought followed by continued summer wetting, conducive for epidemic transmission, last occurred in Florida in 1990. During that year, 226 human cases of SLE were reported throughout the southern half of the state. Since the first appearance of WNV in Florida during 2001, the hydrologic pattern of spring drought followed by summer wetting has not occurred. Because of the similarity of SLEV and WNV transmission cycles (*38*), such a hydrologic pattern will likely result in a major WNV epidemic in the human population of south Florida.

**Figure 1:**
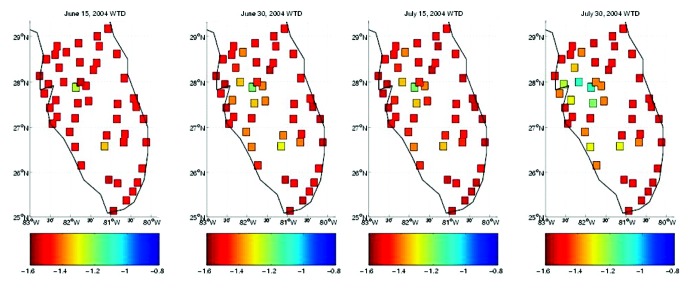
Map of early summer 2004 hydrologic conditions as modeled with the topographically based hydrology model at 49 sites throughout south Florida. Daily averaged conditions are shown for June 15, June 30, July 15, and July 30, 2004. Red shades indicate drier soil conditions, which support less surface pooling; blue shades indicate wetter conditions.

To combat this threat, during 2004 we established real-time monitoring of hydrologic conditions in south Florida. Simulations using the topographically based hydrology (TBH) model (*39**,**40*) were made with National Climate Data Center meteorologic data from 49 station sites in south Florida through the end of 2003 (see [38] for further details). These simulations were then extended in real time during 2004 by using real-time, hourly data of land surface meteorologic conditions distributed at 0.125° resolution available from the National Oceanic and Atmospheric Administration (NOAA) through its Global Energy and Water Cycle Experiment (GEWEX) Continental-Scale International Project Land Data Assimilation System (LDAS) Project. Using these data and the TBH model, we produced maps of land-surface wetness conditions (Figure 1). These maps were made available to personnel at the Florida Medical Entomological Laboratory, as well public health and mosquito control officials throughout Florida. These data were used to evaluate ongoing WNV transmission and the threat of virus transmission to humans. The risk for human infection in Florida was reported in a series of risk maps that were published at http://eis.ifas.ufl.edu and updated as changing conditions warranted. The land surface wetness maps were especially helpful in evaluating WNV transmission in Miami, where sporadic human WNV disease cases were reported from June through September 2004.

**Figure 2:**
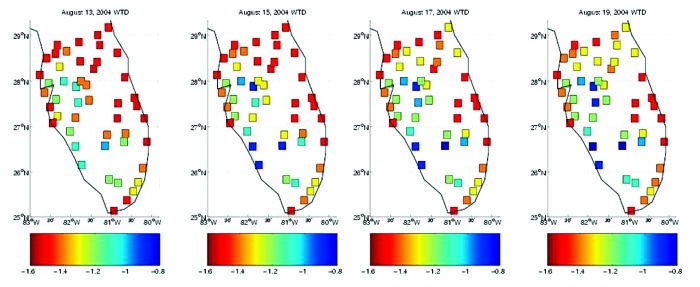
Map of hydrologic conditions during the landfall of Hurricane Charley (August 13) as modeled with the topographically based hydrology model at 49 sites throughout south Florida. Daily averaged conditions are shown for August 13, 15, 17, and 19, 2004. Red shades indicate drier soil conditions, which support less surface pooling; blue shades indicate wetter conditions.

**Figure 3:**
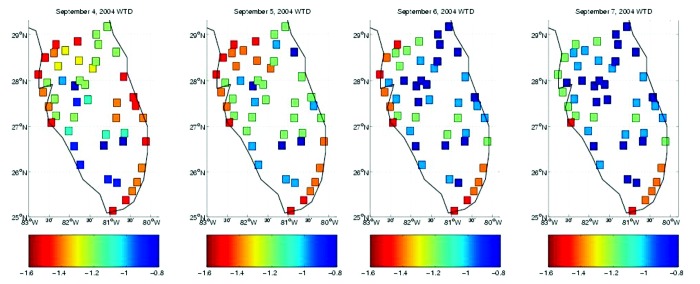
Map of hydrologic conditions during the landfall of Hurricane Frances (September 4) as modeled with the topographically based hydrology model at 49 sites throughout south Florida. Daily averaged conditions are shown for September 4–7, 2004. Red shades indicate drier soil conditions, which support less surface pooling; blue shades indicate wetter conditions.

During 2004, Florida did experience intense, widespread drought; however, the drought persisted through much of the summer. Exceedingly dry conditions existed throughout most of south Florida during late June, July, and early August (Figure 1). This severe drought prevented the infectious mosquito population, which had developed during amplification in May and early June, from dispersing and initiating secondary transmission and amplification foci. This situation resulted in limited WNV transmission to humans in south Florida, where cases were reported in Hillsborough (3 cases), Brevard (4 cases), Broward (3 cases), and Dade (21 cases) Counties. Only with the arrival of Hurricanes Charley and Frances in mid-August and early September (Figures 2 and 3) did land surface conditions in south Florida become considerably wetter. This occurrence, however, was too late to allow the dispersal of infective mosquitoes and the establishment of secondary amplification and transmission foci in time to produce infected mosquitoes on a level that would result in epidemic transmission of WNV.

## Future Prospects and Issues of Scale in Florida

In 2005, we plan to turn operation of this real-time monitoring over to personnel at the Florida Medical Entomological Laboratory. Sustained real-time use of this information will require continual evaluation of TBH model performance, as well as the empiric relationship between modeled land surface wetness conditions and WNV transmission. Our analyses of WNV and SLEV transmission over the last 25 years in south Florida indicate that this empiric relationship is robust and stationary. The spring maps of hydrologic conditions indicate where drought is occurring and WNV amplification is most likely to occur. The summer maps demonstrate where mosquito dispersal and the establishment of secondary transmission foci are likely to occur. The late summer maps indicate where the risk for WNV transmission to humans is greatest in south Florida.

A geographically large-scale WNV epidemic is of primary concern to public health workers in Florida. Large-scale hydrologic events are easily monitored by using records from the current network of meteorologic stations to model land-surface wetness. However, a denser network of stations may be necessary to comprehensively monitor and predict small-scale focal and sporadic WNV transmission in south Florida. To first order, hydrologic conditions in subtropical Florida covary at the spatial scales at which storms organize. With the exception of irrigation, precipitation is the sole source of water to near-surface soils. Because much of the rainfall in Florida is convective, land-surface wetness can vary over short spatial scales (<10 km). The network of meteorologic stations in south Florida is too sparse to detect this small-scale variability. In the future, we plan to use Doppler radar measurements of rainfall and GEWEX LDAS data to run the TBH model at 0.125° resolution throughout south Florida. Such higher resolution monitoring of drought conditions will enable more comprehensive depiction of the small-scale hydrologic variability associated with focal and sporadic WNV amplification and transmission and may enable such events to be detected. Interventions, such as public health warnings or more intense mosquito control efforts, could then be effected at this scale (0.125° resolution).

In addition to monitoring hydrologic conditions, quantitative maps of WNV transmission risk can be constructed at a scale that is not presently available. Risk maps are generated by combining the empirical relationships derived for the dependence of WNV transmission to sentinel chickens and humans on TBH-modeled wetness conditions in south Florida (*38*) with the real-time TBH-model-simulated hydrologic conditions. These predictions provide likelihoods of future WNV transmission to sentinel chickens and to humans. As such, these forecasts do not provide all-or-none predictions, which are right or wrong. Rather, the information allows vector-control experts and public health officials to examine shifts in the likelihood of WNV transmission in both space and time. A cost-benefit analysis might show that a small increase in the likelihood of WNV transmission in an area warrants radical changes in local vector-control efforts. Such an analysis could determine the optimal allocation of public health monies in response to these probabilities. Further investigation of such issues is needed for scientists and public health officials to determine fully the utility and limitations of hydrologic predictions.

Our hope is that an operational WNV and SLEV monitoring system will anticipate the next large arbovirus epidemic in Florida. Results will be even better if timely vector control measures are initiated in response to the real-time modeled hydrologic conditions and avert an epidemic. Whether vector control efforts can mitigate a major WN epidemic remains to be seen; however, vector-control efforts at the front end of an epidemic, during amplification, are far more valuable than control efforts attempted after the epidemic has peaked. The key will be to focus the intervention accurately in space and time to the scale of the problem.
